# Long Interspersed Element–1 (LINE-1): Passenger or Driver in Human Neoplasms?

**DOI:** 10.1371/journal.pgen.1003402

**Published:** 2013-03-28

**Authors:** Nemanja Rodić, Kathleen H. Burns

**Affiliations:** 1 Department of Pathology, Johns Hopkins University School of Medicine, Baltimore, Maryland, United States of America; 2 McKusick-Nathans Institute of Genetic Medicine, Johns Hopkins University School of Medicine, Baltimore, Maryland, United States of America; 3 Sidney Kimmel Comprehensive Cancer Center, Johns Hopkins University School of Medicine, Baltimore, Maryland, United States of America; 4 High Throughput Biology Center, Johns Hopkins University School of Medicine, Baltimore, Maryland, United States of America; Baylor College of Medicine, United States of America

## Abstract

LINE-1 (L1) retrotransposons make up a significant portion of human genomes, with an estimated 500,000 copies per genome. Like other retrotransposons, L1 retrotransposons propagate through RNA sequences that are reverse transcribed into DNA sequences, which are integrated into new genomic loci. L1 somatic insertions have the potential to disrupt the transcriptome by inserting into or nearby genes. By mutating genes and playing a role in epigenetic dysregulation, L1 transposons may contribute to tumorigenesis. Studies of the “mobilome” have lagged behind other tumor characterizations at the sequence, transcript, and epigenetic levels. Here, we consider evidence that L1 retrotransposons may sometimes drive human tumorigenesis.

## Introduction to LINE-1 (L1) Retrotransposons

**Figure 1:**
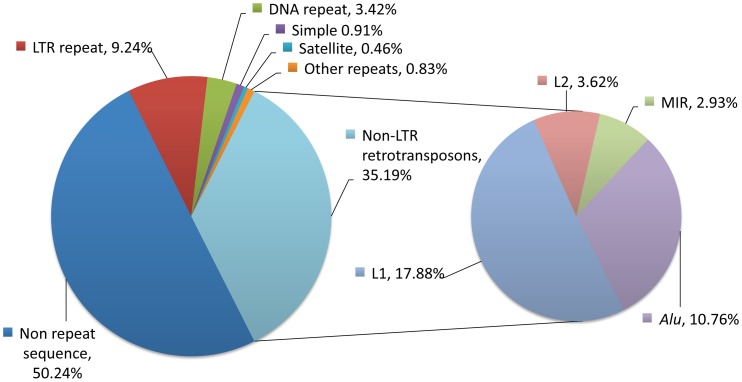
Repetitive sequences in the human genome. About half of our DNA bears homology to known classes of repeats (left chart). The largest class of repeats is the non-LTR retrotransposons, which consists mostly of LINE-1 (L1), L2, MIRs, and Alu elements (right chart). L2 and MIR sequences are not currently active, but subsets of L1 (17.88%), Alu (10.76%), and SVA sequences (not shown, 0.1%) are currently mobile in human genomes and are sources of genetic polymorphisms. Proportions were determined using a RepeatMasker (version rm-20110920, default settings, RepBase sequence database version 16.08) analysis of the Human February 2009 (GRCh37/hg19) assembly. LTR, long terminal repeat retrotransposons; L1, long interspersed element–1; L2, long interspersed element–2; MIR, mammalian wide interspersed repeat; *Alu*, a short interspersed element named for the *Alu*I restriction enzyme; SVA, a composite retrotransposon consisting of short interspersed repeat (SINE-R), variable number tandem repeat (VNTR), and *Alu* like sequence segments.

Repetitive sequences collectively make up greater than half of the human genome and are subdivided into two principal types (Figure 1) [1], [2]. The first is the tandem repeat, or satellite, in which each repeat unit is immediately adjacent to others. Tandem repeat sequences are formed in situ by replication or recombination events [3]. The second type consists of interspersed repeats, which are repeated sequences that are distributed throughout the genome rather than occurring in tandem [4]. Interspersed repeat sequences are derived from transposable elements or mobile DNAs, further described as either DNA or RNA transposons, depending on the mechanism of their spread. While DNA transposons use a “cut-and-paste” mechanism, RNA transposons use a “copy-and-paste” mode of moving in genomes. RNA transposons use an RNA intermediate and are also referred to as retrotransposons, retroposons, or retroelements; we will use the term *retrotransposons* in this review. The only active mobile DNAs in modern-day humans are the autonomous L1 retrotransposon and non-protein-coding (nonautonomous) sequences its machinery mobilizes.

**Figure 2:**
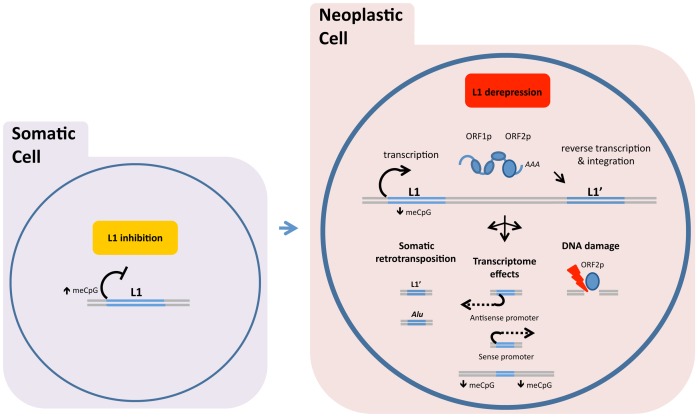
DNA methylation and related mechanisms inhibit LINE-1 (L1) expression, and hypomethylation of DNA allows the L1 retrotransposon “life cycle” to proceed. In normal somatic cells, DNA methylation and related mechanisms inhibit LINE-1 (L1) expression (left image). In neoplastic cells, hypomethylation of DNA allows the L1 retrotransposon “life cycle” to proceed (right image). Retrotransposition is shown in a simplified schematic under the red box as (from left to right) transcription, assembly of ORF1p and ORF2p with L1 RNA, and insertion of a new L1 sequence (L1′). Related tumor effects are conceptually shown as (i) somatic retrotransposition of L1 and nonautonomous repeat elements, such as *Alu* repeats; (ii) transcriptional changes induced by L1-encoded promoters (in antisense and sense) or impacts on area methylation; and (iii) L1 ORF2p-generated DNA breaks. ASP, L1 antisense promoter.

The L1 life cycle entails three steps (Figure 2, red box). The first step is transcription of a genomic L1 into RNA, which is mediated by RNA polymerase II from an internal L1 promoter. Transcription from an internal *anti*sense L1 promoter may occur concurrently. In the second step, the RNA is translated into two L1-encoded proteins: ORF1p, an RNA-binding protein, and ORF2p, a protein with reverse transcriptase and endonuclease activities. These proteins associate with the L1 transcript, and the resulting ribonucleoprotein (RNP) complexes are then transferred to the nucleus. The third step is termed target-primed reverse transcription (TPRT). In the course of TPRT, ORF2p cleaves the target DNA (often at a 5′-TTTTAA-3′ consensus sequence) and uses the 3′ hydroxyl group to prime the reverse transcription reaction. Synthesis of the second strand and resolution of the structure are poorly understood. Because the L1 life cycle generates DNA breaks, cell host proteins that mediate DNA repair are likely involved.

In the human genome, the majority of our estimated 500,000 L1 copies are (1) present on both homologous chromosomes, (2) truncated at the 5′ end (mean length, 0.9 kb), and (3) incapable of encoding ORF1p and ORF2p and transposing. A relatively small number are potentially active, full-length L1 elements (approximately 6 kb long) with intact coding sequences for ORF1p and ORF2p [5]. Full-length L1 insertions largely reflect the activity of the Ta1 subfamily of human-specific L1. Functional human-specific L1 insertions passed in the germline have deleterious effects on fitness and are hence under negative selection but continue to be a source of genetic diversity.

In this review, we discuss inhibition of L1 retrotransposition in normal somatic cells and activation of L1 in cancer cells. We also consider possible causal roles of L1 in tumorigenesis, discussing ways in which it may influence regulation of host genes or genomic stability apart from the canonical transposition pathway.

## Inhibition of L1 Retrotransposition in Normal Germline and Somatic Cells

L1 is regulated by distinct pathways in different cell contexts. In the male germline, L1 is inhibited via an elaborate system involving Piwi-interacting RNAs (piRNAs) that ultimately methylates genomic L1 sequences. This depends on methylation regulator DNMT3L [6] and PIWIL4 (also known as MIWI2) [7], as well as PIWI proteins involved in piRNA production. In embryonic stem cells, which can be used to model chromatin regulation in preimplantation-stage embryos, inherited L1 methylation is maintained by DNA methyltransferases DNMT1, DNMT3A, and DNMT3B [8]. In embryonal carcinoma cell lines, which are sometimes used for the same purpose, newly retrotransposed L1 sequences are silenced by histone alterations, including deacetylation of H4 and dimethylation of H3K9 [9].

In addition to the host factors mentioned above, other proteins have been implicated in L1 repression in various somatic tissues. These include methyl CpG binding protein 2 (MECP2) [10], lymphoid-specific helicase (HELLS) [11]–[13], a retinoblastoma protein-containing complex (RB1) [14], the 3′ repair exonuclease 1 (TREX1) [15], excision repair cross complementing 1 (ERCC) [16], and apolipoprotein B mRNA editing enzyme, catalytic proteins APOBEC3A, APOBEC3B, and APOBEC1 [17], [18]. In aggregate, these and other proteins seem to prevent L1 expression or somatic retrotransposition in all normal tissues except for the developing brain [19], [20]. However, the relative importance of each of these proteins to transposon silencing in the breadth of human cell types remains to be fully characterized.

## L1 Retrotransposition in Neoplasms

Somatic L1 insertions have the inherent potential to drive tumorigenesis by activating oncogenes or inactivating tumor suppressor genes. However, somatic L1-related rearrangements that have driven tumorigenesis have rarely been discovered in humans. Before 2010, only two such cases had been reported. The first case involved a gene-activating rearrangement at the *MYC* oncogene locus in breast ductal adenocarcinoma [21]. The second case involved a newly integrated L1 sequence that inactivated the APC tumor suppressor gene in colon cancer cells. This insertion had sequence features characteristic of an L1 retrotransposition [22]. The scarcity of somatic L1 insertions that have been found to drive tumorigenesis may be owed in part to the limitations of traditional molecular assays, which have not lent themselves to identifying de novo L1 insertions.

The introduction of targeted next-generation sequencing and analysis methods in recent years has led to additional reports of somatic L1 retrotransposition in colon cancer [23], [24], as well as in lung [25], prostate [24], and ovarian [24] carcinomas. In one study, researchers targeted insertion sites for sequencing and found nine tumor-specific somatic L1 insertions in six of 20 primary lung carcinomas tested [25]. In another study, Lee and colleagues [24] surveyed whole genome sequences from a variety of tumors included in The Cancer Genome Atlas project and discovered 183 L1 insertions in colorectal, prostatic, and ovarian carcinomas. The number of insertions per tumor ranged from an average of four in several ovarian carcinoma specimens to 106 in a single colon carcinoma specimen. All of the insertions were severely truncated at the 5′ end, perhaps reflecting inhibition of TPRT by robust somatic cell mechanisms.

The fact that the somatic insertions reported by Lee and colleagues were skewed toward genes that are commonly downregulated or mutated in the tumors that they studied suggests that L1 insertions may contribute to cancer formation. In order to determine whether L1 actually does so, it would help to identify specific loci that are recurrently disrupted by L1 insertions. Ongoing work to profile L1 positions in additional tumor types, metastatic samples, and samples of disease relapse after therapy may lead to the identification of such loci.

## L1 Expression in Human Neoplasms

Whereas normal adult tissues do not express L1 ORF1p [26], [27], selected human neoplasms do express both L1 RNA and proteins. Several epithelial neoplasms, including renal, ovarian, lung, and prostate carcinomas, express L1 RNA at detectable levels [28]. This L1 derepression can be associated with poor clinical features. For example, in pancreatic ductal adenocarcinomas, higher levels of L1 RNA correlate with higher grade lesions and poorer clinical outcomes [29]. Similarly, in high-grade breast carcinomas, higher levels of nuclear L1 ORF1p protein, as determined by immunohistochemistry, correlate with poorer clinical outcomes [26].

In addition to these definitive cases of L1 expression, there have been other cases indicating L1 derepression. For example, L1 promoter hypomethylation has been reported in multiple myeloma [30], chronic myeloid leukemia (CML) [31], and chronic lymphocytic leukemia, suggesting that L1 might be transcribed in these cancers [32]. Of note, CML cases with L1 promoter CpG dinucleotide hypomethylation tend to be aggressive neoplasms leading to poor prognosis, although whether L1 is causally related to the aggressiveness is not known [31].

L1 RNA expression, ORF1p expression, and L1 methylation status are more readily detected than ORF2p expression using current reagents. While it is plausible that ORF2p expression also occurs in cases exhibiting the first three features, it is possible that examples of decoupled regulation will be described. ORF2p expression is a major mechanism, in addition to retrotransposition, through which L1 may impact the genome. Cell-culture-based studies convincingly show that ORF2p expression can be mutagenic, inducing not only mutations associated with canonical retrotransposition events but also DNA breaks [33]–[35] and large genomic deletions [36]. ORF2p has also recently been implicated in recurrent DNA translocations that occur in conjunction with other DNA-binding proteins that may target its endonuclease activity within the genome [37].

## L1 and Antitumor Effects of Reverse Transcriptase (RT) Inhibition

Several nucleoside analogues have been shown in biochemical analyses to inhibit the RT activity of ORF2p and, in cell-culture-based retrotransposition assays, to reduce the number of L1 retrotransposition events [38], [39]. Nucleoside analogues function by becoming incorporated into growing DNA strands, where they act as chain terminators. In contrast, other types of RT inhibitors, known as nonnucleoside RT inhibitors (NNRTIs), function by binding to a “pocket” of the RT enzyme, and may be more restricted in their scope of activity. NNRTIs are much less potent than nucleoside analogues, or do not affect ORF2p activity at all in retrotransposition assays. For example, efavirenz, an NNRTI antiretroviral drug, affects ORF2p activity only at very high concentrations, and nevirapine, also an NNRTI antiretroviral drug, has negligible impact on ORF2p activity.

In cell-culture-based cancer models, both nucleoside RT inhibitors and NNRTIs have been shown to promote senescence and differentiation, and reduce invasive growth. For example, the nucleoside analog abacavir inhibits the proliferation and migration of a prostate cancer cell line [40], and the NNRTIs efavirenz and nevirapine inhibit the growth of malignant melanoma and prostate cancer cell lines in a dose-dependent manner [41]. Nevirapine also inhibits the growth of teratocarcinoma, colon carcinoma, lung carcinoma, and acute myeloid leukemia cell lines in a dose-dependent manner [42]. Whether L1 RT activity is relevant to these observations is hotly debated. Some or all of these drug effects may be mediated through pathways other than inhibition of L1 RT.

## Impacts of L1 on Transcription Initiation in Cancer

In some cases, transcription is initiated within an L1 to form a chimeric mRNA that contains L1 sequence (sense or antisense) and downstream exons of a host gene. In fact, in normal mouse tissues, transcription is initiated frequently within L1 sequence, as Faulkner et al. determined by sequencing the 5′-most nucleotides of RNAs from both normal and neoplastic tissues, using cap-analysis gene expression tag technology (CAGE) [43]. Transcription initiation often occurs within 5′ L1 sequence, which contains both sense and antisense promoter activities. The CAGE analysis also revealed highly specific patterns of transcriptional activity from L1 across samples. In human cancers, aberrant expression of chimeric transcripts may play important roles in tumorigenesis. Investigators have noted transcript variants initiated by L1 antisense promoters in bladder carcinoma [44], chronic myeloid leukemia (CML) [31], esophageal adenocarcinoma [45], and breast carcinoma [46].

L1 transcription initiation can be drug induced. For example, Weber et al. showed that transcription of the L1-cMet sequence, which originates within the *MET* proto-oncogene locus, is specifically induced in CML cells by the demethylating agents azacytidine and decitabine [47]. Their work further indicated that L1-cMet functions as a potential tumor suppressor by interfering with expression of the cMet transcript, ultimately leading to decreased cMet signaling. The fact that L1 derepression is associated with tumor suppression in some cases and cancer progression in others, as mentioned earlier, makes us cognizant that the roles of L1 derepression in cancer are complex.

L1 insertions may also influence transcription initiation from the canonical transcriptional start site of a gene locus. Estecio et al. found evidence of this by noting an increased number of L1 insertions near selectively *hypomethylated* transcriptional start sites of single-copy genes in cancer cell lines [48]. Thus, epigenetic dysregulation of L1 sequences may promote transcription of nearby oncogenes.

## Conclusions

There is growing evidence that L1 expression and even complete retrotransposition occur in selected human cancers, suggesting that L1 may drive tumorigenesis. Even if L1 does not drive tumorigenesis through new insertions or ORF2p genetic damage, L1 sequences are such prevalent genomic passengers that they are likely to contribute to gene expression during tumor development, and affect responses of tumors to treatment. New reagents, experimental approaches, and informatics tools are being developed to provide more complete pictures of L1-mediated roles in human neoplasms.
